# Physical Therapy Provision After COVID-19 in the Netherlands: Retrospective Cohort Study

**DOI:** 10.2196/88633

**Published:** 2026-09-28

**Authors:** Renee Veldkamp, Peter Spreeuwenberg, Arie Cornelis Verburg, Thomas Johannes Hoogeboom, Rob A de Bie, Willemijn M Meijer

**Affiliations:** 1 Nivel, Netherlands Institute for Health Services Research, Otterstraat 118, Utrecht, 3513CR, The Netherlands, 31 302729700; 2 Radboud Institute for Health Sciences, IQ Health, Radboud University Medical Center, Nijmegen, The Netherlands; 3 Association for Quality in Physical Therapy (SKF), Zwolle, The Netherlands; 4 Department of Rehabilitation, Physiotherapy Science and Sports, UMC Utrecht Brain Center, University Medical Center Utrecht, Utrecht, The Netherlands; 5 Research Group Innovation of Human Movement Care, University of Applied Sciences Utrecht, Utrecht, The Netherlands; 6 CAPHRI School for Public Health and Primary Care, Faculty of Health, Medicine and Life Sciences, Maastricht University, Maastricht, The Netherlands

**Keywords:** COVID-19, physical therapy, electronic health records, routine health care data, Dutch primary care

## Abstract

**Background:**

Many patients have been treated by physical therapists after an infection with COVID-19. The policy changes and unique situation of the COVID-19 pandemic necessitate a retrospective analysis.

**Objective:**

This study examined physical therapy provision for adult patients recovering from COVID-19 in primary care in the Netherlands over the first 2.5 years of the pandemic. The primary aim was to examine changes in the amount of health care provision and associated factors. Furthermore, we aimed to gain insight into the patient population and changes over time, as well as the use of advised outcome measures in physical therapy after COVID-19.

**Methods:**

We conducted a retrospective cohort study using routine health care data from the electronic health records of physical therapy practices in Dutch primary care. In total, 71,152 COVID-19 treatment trajectories initiated between March 1, 2020, and December 18, 2022, were included. Multilevel generalized linear mixed model analyses examined changes over time in the patient population and in the amount of physical therapy within treatment trajectories (ie, duration in weeks and number of consultations). Outcome measures were analyzed using descriptive statistics.

**Results:**

Completed treatment trajectories had an overall duration of 24 weeks with 28 consultations, with a decreasing trend in the amount of physical therapy provided over time (*P*<.001). Also, a parabolic effect of age (*P*<.001) was found, showing a maximum number of consultations and duration of the treatment trajectory among older patients aged 60 to 79 years, with smaller quantities for younger and older patients. Treatment trajectories for women were 1.5 weeks longer than those for men (β 1.5, SE 0.12; *P*<.001) but did not include more consultations (*P*=.76). In 83% (59,184/71,152) of treatment trajectories, a baseline score on at least one of the included advised outcome measures was registered. Median (IQR) baseline scores were 420 (340-490) m on the 6-minute walking test (n=27,035); 13 (10-17) seconds on the 5-times sit-to-stand test (n=2337); 5.8 (5‐6.3) points on the Fatigue Severity Scale (n=995); 8 (7-10) points on the Patient-Specific Functional Scale (PSFS; n=55,074); 10 (9-12) points on the Short Physical Performance Battery (n=4665); and 29 (22-38) and 30 (24-40) kg of grip strength, respectively, on the left (n=12,306) and right (n=12,293) hand. Difference scores could be examined for the 6-minute walking test, grip strength, and the PSFS.

**Conclusions:**

This study demonstrates the potential of routinely collected health care data to describe real-world physical therapy provision for COVID-19 in Dutch primary care. Patients showed severe perceived fatigue, reduced functional capacity, and lower-limb strength at the start of physical therapy. The amount of physical therapy declined over the first 2.5 years of the pandemic, differed by age and gender, and was generally lower than the amount reimbursed by the Dutch government through basic health insurance.

## Introduction

Since the start of the SARS-CoV-2 pandemic in early 2020, millions of people have contracted COVID-19 [1]. Patients who were admitted to the hospital could develop acute respiratory distress syndrome, muscle weakness, or post–intensive care syndrome [2-4]. Furthermore, it is estimated that between 3% and 6% of people continue to experience symptoms beyond the acute phase of the infection, known as postacute COVID-19 syndrome [5-8]. Common persistent symptoms include fatigue, dyspnea, memory problems, posttraumatic stress disorder, sleep disorders, anxiety, depression, and joint pain [9-11], substantially impacting general health, activities of daily living, quality of life, and working capacity of patients [9,12-14]. Considering the impact of COVID-19 and postacute COVID-19 syndrome on motor and cognitive functions, many patients have been treated by allied health care providers, mostly by physical and exercise therapists (hereafter, physical therapists) [15]. Physical therapists play an important role in supporting respiratory function, guiding active mobilization, ensuring a good load vs load capacity [16], and providing therapy for impairments in movement-related functioning affecting daily activities or participation [17].

Therefore, a temporary policy change was introduced in the Netherlands during the early months of the pandemic to enhance the accessibility to physical therapy after a COVID-19 infection under certain conditions [18]. Usually, in the Netherlands, physical therapy is included in the obligatory basic health insurance package only for a selection of diagnoses. Physical therapy for other diagnoses is paid out-of-pocket or reimbursed through voluntary supplementary health insurance. As of July 18, 2020, basic health insurance temporarily covered up to 50 physical therapy sessions for patients with COVID-19 over a 6-month period, with the option for a medically justified extension [18]. Furthermore, as COVID-19 was a new condition, treatment guidelines had to be developed based on the experiences of the first patients with COVID-19 and the opinions of experts. Over time, many studies have provided more insight into the biological and clinical aspects of the condition, and several adjustments to treatment guidelines were made [17]. However, it is not known how the policy regarding physical therapy for COVID-19 in primary care and the changing circumstances over time influenced the physical therapy use of patients with COVID-19. With such policy changes and unique circumstances, it is necessary to look back at how this was handled in order to learn from them and increase future pandemic preparedness.

Therefore, the overall aim of this study was to gain insight into the real-world physical therapy provision for adult patients recovering from COVID-19 in Dutch primary care over the first 2.5 years of the pandemic. In the Netherlands, several registries are available that receive routine health care data from the electronic health records (EHRs) of primary care physical therapy practices. Data from these registries can be used to build a retrospective cohort of the patients who sought physical therapy for symptoms of COVID-19, using real-world data. These data therefore offer the opportunity to look back on the period of the COVID-19 pandemic and learn from it. More specifically, the primary aim of this study was to examine the amount of physical therapy provided for patients after a COVID-19 infection over time and to identify which factors (ie, patient characteristics and course of the pandemic) were associated with it. The second aim was to provide insight into changes over time in the patient population starting with physical therapy after COVID-19 and in the clinical profile of these patients. The final aim was to examine the use of outcome measures as recommended in Dutch guidelines concerning physical therapy for COVID-19 [17].

## Methods

### Study Design and Setting

This retrospective cohort study was set up as part of a greater nationwide project in the Netherlands to evaluate the recovery of patients receiving allied health care treatment in primary care practices after a SARS-CoV-2 infection, the ParaCOV study (The Dutch Consortium Allied Healthcare COVID-19) [19]. Routine health care data from EHRs collected in the databases of (1) Nivel, the Netherlands Institute for Health Services Research, Primary Care Database (Nivel-PCD) [20], (2) the Dutch National Data Registry (in Dutch: LDK) of the Dutch Association for Quality in Physical Therapy (in Dutch: SKF), (3) the National Registry Exercise Therapy (in Dutch: LDO), and (4) the National Physical Therapy Database (in Dutch: LDF) of the Royal Dutch Society for Physical Therapy (in Dutch: KNGF) were combined. Together, these databases contain information on approximately 1 million unique patients receiving physical therapy in primary care for a broad range of diagnoses. The data in these registries are structured in the same way, enabling a smooth process of combining the datasets. To avoid duplicates, data from physical therapy practices affiliated with data collection via Nivel (Nivel-PCD, LDK, and LDO), which are all collected via the same route, were used as a starting point and supplemented with data from nonoverlapping practices affiliated with LDF. To define comorbidities (see *Outcomes*), data from approximately 500 general practices collected in Nivel-PCD, covering around 10% of the Dutch population, and the Nivel-PCD physical therapy practices were linked for the subset of patients for whom information was available in both of these databases.

### Ethical Considerations

Data collected for the registries were pseudonymized at the source (within the practices), leaving out direct identifying information such as names and address. This study has been approved in accordance with the governance code of Nivel-PCD under number NZR-00322.046. The use of EHRs for research purposes is allowed under certain conditions. When these conditions are fulfilled, neither obtaining informed consent from patients nor approval by a medical ethics committee is obligatory for this type of observational study containing no directly identifiable data (Art 24 GDPR [General Data Protection Regulation] Implementation Act jo art 9.2 sub j GDPR [21]).

### Cohort

In the EHRs of physical therapists, data on the provided care and accompanying information are registered per patient and per diagnosis in a so-called treatment trajectory, wherein the diagnosis is registered using the diagnostic code for allied health care professionals (DCSPH-code [22]). Inclusion criteria for the cohort of physical therapy treatment trajectories in this study were (1) DCSPH-code 9363 (ie, COVID-19); (2) starting March 1, 2020, or later; (3) consisting of at least 2 consultations, that is, registered contacts on separate dates (see also *Outcomes*); and (4) adult patients (18 y and older). Physical therapists were only allowed to use this diagnostic code when a patient had a referral from a general practitioner or medical specialist indicating that the patient was in need of physical therapy after a COVID-19 infection. Data were available up to February 20, 2023. However, to reduce the chance of missing treatment trajectories based on not yet available data in the respective databases and to avoid the drop in starting treatment trajectories over the Christmas holidays, only treatment trajectories starting the latest on December 18, 2022, were included in the final cohort.

Additionally, the study period was divided into start weeks, running from Monday to Sunday, with March 1, 2020, falling into start week 1 and the week of December 12 to 18, 2022, being the last start week (start week 147). Each treatment trajectory was assigned to the week in which that treatment trajectory started, based on the date of the first consultation.

The amount of physical therapy provided can only be analyzed on treatment trajectories that have ended. Therefore, for those analyses, a subset of treatment trajectories was defined. A treatment trajectory was defined as being closed when the therapist registered it as such or when there had not been a consultation for more than 60 days. Based on the median duration of closed treatment trajectories in the full dataset, treatment trajectories starting in the last 22 start weeks of the final cohort were excluded to minimize the effect of including only shorter closed treatment trajectories near the end of the data collection. Therefore, this subset consisted of closed treatment trajectories starting the latest in start week 134 (September 18, 2022).

Data are provided per treatment trajectory, and the assumption was made that all treatment trajectories could be assigned to individual patients (as 95.4%, 64,668/67,825 of patients had only 1 registered treatment trajectory over time).

### Outcomes

#### Patient Characteristics

Patient characteristics, such as “age,” “gender,” “duration of symptoms,” and “number of comorbidities,” were collected at the start of treatment. “Duration of symptoms” was registered by the physical therapist at the start of the treatment trajectory and refers to the duration in weeks from the start of the symptoms to the first visit of the patient at the physical therapist in three categories: (1) first visit within 0 to 2 weeks, (2) first visit within 3 to 12 weeks, and (3) first visit more than 12 weeks after the start of the symptoms for which the patient visits the physical therapist. The number of comorbidities (0, 1, or more than 1) was defined for the subset of patients for whom data were available from (1) Nivel-PCD general practices and/or (2) registered outcome measures in the treatment trajectory. Per treatment trajectory, it was determined whether the patient was registered as having diabetes, obesity, heart failure, cardiovascular diseases and hypertension, chronic pulmonary disease, chronic kidney damage, and liver disease, in at least one of the data sources, or none of those comorbidities in either source. See Multimedia Appendix 1 for the definition of the comorbidities.

#### Population Characteristics

The subtype of the SARS-CoV-2 infection was unknown at the individual patient level but was preassigned to each treatment trajectory based on the most dominant virus subtype at the population level 9 weeks before the treatment start. The most dominant virus subtype was defined as the virus type that had the highest percentage of patients in that week, according to the Dutch National Institute for Public Health and the Environment (in Dutch: RIVM [23]). A delay of 9 weeks before the start of the treatment trajectory was chosen based on the median time between the start of the infection and the start of the physical therapy treatment trajectory, as defined in the data from the national prospective cohort study on allied health care in patients recovering from COVID-19 [19].

#### Amount of Health Care Provision Per Treatment Trajectory

The provided health care per treatment trajectory is registered as so-called care products. A consultation was defined as a date with a registered care product within the treatment trajectory, irrespective of the number of care products registered within the treatment trajectory on that specific date. For example, when 2 care products (eg, an “intake” and “treatment”) for a patient were registered on the same date in the same treatment trajectory, this counted as one consultation. Based on this, the variables were defined as follows (Figure 1):

- Start date: The first date in the treatment trajectory with a consultation (Figure 1A). An exception was made for treatment trajectories in which there was a gap of more than 60 days between the first and second consultations, while after the second consultation, there were more consultations within less than 60 days. In that case, the second consultation was taken as the start date of the treatment trajectory, assuming that the actual treatment started then (Figure 1B).
- Last date: The last date in the treatment trajectory with a consultation (Figure 1A), unless there was a gap of more than 60 days since the penultimate consultation, while the penultimate consultation was part of a streak of consultations (Figure 1B). Here, the assumption was made that a sole registered care product after 60 days was not part of the actual treatment but had more administrative or aftercare reasons.
- Duration of the treatment trajectory: The number of weeks between the assigned start date and the last date of the treatment trajectory (the bold line in Figure 1).
- Number of consultations: The number of consultations within the determined treatment trajectory. In the final cohort, only treatment trajectories consisting of at least 2 consultations were included.

**Figure 1.**
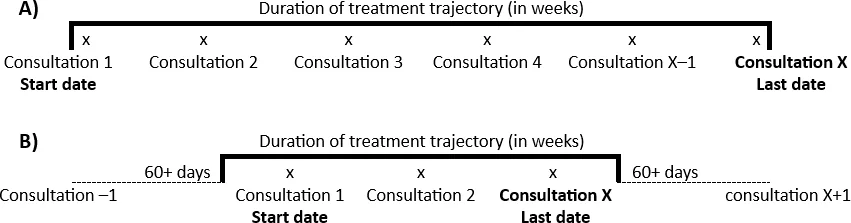
Visualization of determined variables regarding physical treatment trajectories for COVID-19: start date, last date, number of consultations within, and duration in weeks of a treatment trajectory. The treatment trajectory is determined based on the registered consultations. The first consultation defines the start date and the last consultation, the last date of the treatment trajectory (A), in which first and last consultations that are separated by at least 60 days from the respective next and previous consultations are not considered (B). The duration of the treatment trajectory and the number of consultations within it are the number of weeks between the start date and last date (bold line) and the total number of consultations (consultation X).

#### Outcome Measures

Many different outcome measures can be registered in the EHRs and sent to registries [24]. Therefore, a selection of outcome measures was based on a combination of the position statement provided by the KNGF [17], guidelines for physical therapists provided by the ParaCOV-consortium [19], and data availability in the registries [25]. This concerned 4 functional therapist-assessed outcome measures and 2 patient-reported outcomes. The 5-times sit-to-stand test (5TSTS) was recommended for functional strength. In the 5TSTS, the patient is asked to get up and sit down in a chair 5 times in a row as fast as possible without using their hands [26]. The 6-minute walking test (6MWT) and Short Physical Performance Battery (SPPB) were (optionally) recommended for functional capacity. The 6MWT measures the maximum distance a patient can walk within 6 minutes [27]. The SPPB consists of 3 parts: balance, gait speed, and 5TSTS, on which 0 to 4 points can be scored [28]. The 5TSTS is performed as described above, and the gait speed task consists of walking 4 meters at a normal pace; in both subtasks, the time is recorded and scored. The balance tests include, in this particular order, side-by-side stand, semitandem stand, and tandem stand. Grip strength was recommended to be measured with a hand-held dynamometer as an indicator of overall upper-body strength [29].

Patient-reported outcomes were the Fatigue Severity Scale (FSS) for patient-reported fatigue [30] and the Patient-Specific Functional Scale (PSFS) for functional status [31]. In the PSFS, the patient is asked to rate the extent to which they feel limited in 3 self-selected activities that are most important to them. These activities should, according to the patient, be (1) relevant, (2) limiting performance, and (3) regularly performed (at least weekly). Here, the first one registered was taken as an outcome.

Lastly, the Global Perceived Effect (GPE) was also included as a measure of the perception of the patient regarding recovery and/or satisfaction with the treatment [17,32].

Measurements were considered to reflect baseline data when registered in the period from 2 weeks before the start date, as defined above, until 1 week (for treatment trajectories with a duration of 2‐4 wk) or 2 weeks (minimum duration of 5 wk) after the start date. Similarly, measurements were considered to reflect the posttest when registered in the period from 1 to 2 weeks, depending on the duration of the treatment trajectory, before the last date of the treatment trajectory until 2 weeks after the last date. Difference scores were calculated for each outcome measure by comparing baseline and posttest values, when at least 1000 treatment trajectories were registered with that outcome measure. The threshold of 1000 treatment trajectories was set arbitrarily to ensure a substantial sample size, given the secondary use of the data over an extended study period. If grip strength of both hands was measured, the difference score on the hand with the greatest difference score was used.

### Analysis

All data preparation and descriptive analyses were conducted in Stata (version 16; StataCorp LLC). Missing values were not imputed, and the significance level was set at 0.01, given the considerable size of the dataset. Descriptives of the total cohort were assessed by providing means and SDs, or frequencies and percentages, as appropriate. Statistical analyses were conducted in MLwiN (Centre for Multilevel Modelling, University of Bristol).

#### Patient Population Starting Physical Therapy for COVID-19 Over Time and Clinical Profile

Multilevel generalized linear mixed model analysis was conducted to examine the patient population starting physical therapy within the cohort over time (relative incidence). Defined levels were physical therapy practices, patients, and start week.

Relative incidence was determined as the proportion of treatment trajectories starting per age subgroup and gender subgroup in that start week, compared to all starting physical treatment trajectories for COVID-19 in primary health care in the Netherlands in the first 2.5 years of the pandemic. Notably, it does not give the incidence of patients starting physical therapy for COVID-19 in the Dutch population, as there is no underlying population available in the registries for persons who did not attend an associated physical therapy practice. In the multilevel analysis for relative incidence, the dichotomous incidence outcome was analyzed as logistic regression, and all start weeks (weeks 1 to 147 [March 1, 2020, to December 18, 2022]) in the final cohort were included. Gender (man and woman), age (18‐39, 40‐59, 60‐79, and >80 y), and time (centered around the midpoint of the time range) as a polynomial of the third-order (to capture the nonlinear curve over time) were combined. The combination of gender and age (8 categories in total) estimated a separate time effect for each of the 8 categories. Virus type (ie, original Wuhan, Alpha, Delta, and Omicron) was included as a fixed effect.

To examine the clinical profile of patients at the start of the treatment trajectory, the median and IQR of baseline scores on the selected outcome measures were examined.

#### Changes in Physical Therapy Provision for COVID-19 Over Time

Multilevel generalized linear mixed model analyses were also executed to examine the amount of physical therapy provided over time by (1) the number of consultations within closed treatment trajectories and (2) the duration of closed treatment trajectories in weeks. The defined levels were physical therapy practices, patients, and start week.

The variables for the amount of physical therapy provided, that is, the number of consultations in and the duration in weeks of a treatment trajectory, were analyzed as normal regressions on the subset of closed treatment trajectories. In these multilevel analyses, only closed treatment trajectories with start weeks 1 to 134 (March 1, 2020, to September 18, 2022) were included. In these 2 models, gender, age, and virus type were included as fixed effects. The same multilevel analyses were conducted on the subset of closed treatment trajectories wherein the number of registered comorbidities could be determined, while including the number of comorbidities (0, 1, and >1) as a fixed effect.

#### Use of Recommended Outcome Measures

Analyses of the use of recommended outcome measures were performed on the subset of closed treatment trajectories. To examine the use of the outcome measure in the closed treatment trajectories, the percentage of treatment trajectories, including the outcome measure as a baseline measure or as both baseline and posttest, was calculated. Furthermore, the median and IQR of the scores at baseline, at posttest, and of the difference score were calculated for each outcome measure. Difference scores less than the 25th percentile minus 1.5 times the IQR or greater than the 75th percentile plus 1.5 times the IQR were treated as outliers.

## Results

### Cohort Description

Figure 2 provides a flowchart of the included treatment trajectories, and Table 1 provides the descriptives of the cohort. In the final cohort, a total of 71,152 new treatment trajectories for COVID-19 were included. Most treatment trajectories started with the original variant as the dominant virus type (n=21,564, 30.3%), followed by Omicron (n=18,611, 26.2%), Delta (n=16,982, 23.9%), and Alpha (n=13,995, 19.7%) variants. In one of the treatment trajectories, the gender of the patient was missing. A total of 66,653 treatment trajectories were considered closed and thus included in the specific analyses of the number of consultations in, and the duration of, closed treatment trajectories (Figure 2). For the subset of closed treatment trajectories of patients for whom comorbidities could be determined (n=5307), 65.3% (n=3466) had no registered comorbidities, 24.0% (n=1275) had one comorbidity, and 10.7% (n=566) had multiple comorbidities.

**Figure 2.**
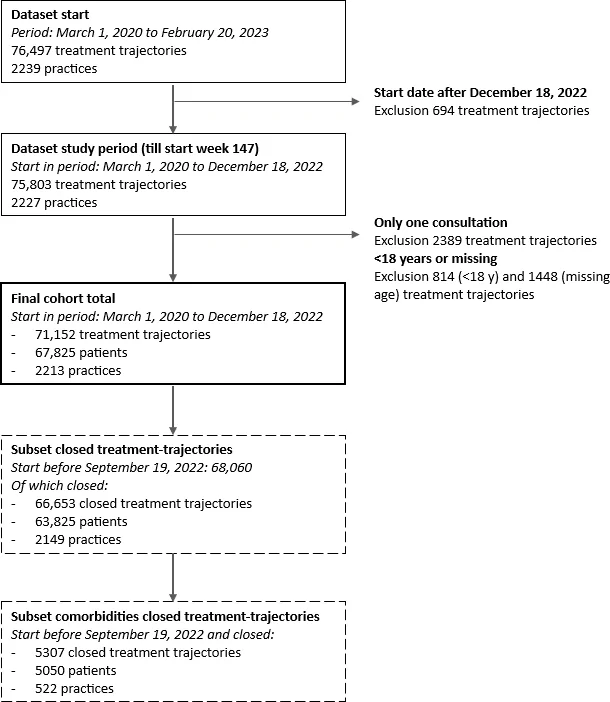
Flowchart of included physical therapy treatment trajectories for COVID-19 in primary care in the Netherlands during the period 2020 to 2022 and their subsets.

**Table 1.** Descriptives of the included patients and physical therapy treatment trajectories for COVID-19 in the retrospective cohort over the years 2020 to 2022.

	Total cohort	Women	Men
Treatment trajectories, n (%)	71,152 (100)	45,478 (63.9)	25,673 (36.1)
Unique patients, n	67,825	43,253	24,571
Dominant virus type, n (%)			
Original	21,564 (30.3)	13,698 (30.1)	7866 (30.6)
Alpha	13,995 (19.7)	8322 (18.3)	5673 (22.1)
Delta	16,982 (23.9)	10,873 (23.9)	6108 (23.8)
Omicron	18,611 (26.2)	12,585 (27.7)	6026 (23.5)
Age, mean (SD)	52.2 (15.4)	49.9 (15.1)	56.2 (15.2)
Age group (y), n (%)			
20-39	15,511 (21.8)	11,684 (25.7)	3827 (14.9)
40‐59	33,058 (46.5)	22,064 (48.5)	10,994 (42.8)
60‐79	19,541 (27.5)	10,251 (22.5)	9290 (36.2)
>80	3041 (4.3)	1479 (3.3)	1562 (6.1)
Duration of symptoms at start of treatment (w), n (%)^a^			
0‐2	6271 (9.7)	3787 (9.2)	2484 (10.7)
3‐12	37,550 (58.3)	23,898 (57.9)	13,651 (58.8)
>12	20,611 (32.0)	13,526 (32.8)	7085 (30.5)
Baseline scores			
6MWT^b^ (m)			
Score, median (IQR)	420 (340-490)	410 (340-480)	430 (346-504)
Count, n	27,035	17,339	9696
5TST^c^ (s)			
Score, median (IQR)	13 (10-17)	13 (10-17)	13 (10-17)
Count, n	2337	1453	884
SPPB^d^ (points)			
Score, median (IQR)	10 (9-12)	10 (9-12)	10 (8-12)
Count, n	4665	2961	1704
PSFS^e^ (points)			
Score, median (IQR)	8 (7-10)	8 (7-10)	8 (7-10)
Count, n	55,074	35,060	20,014
FSS^f^ (points)			
Score, median (IQR)	5.8 (5‐6.3)	5.8 (5.1‐6.3)	5.7 (4.9‐6.3)
Count, n	995	652	343
GS^g^: left (kg)			
Score, median (IQR)	29 (22-38)	26 (20-30)	40 (32-49)
Count, n	12,306	7864	4442
GS: right (kg)			
Score, median (IQR)	30 (24-40)	27 (21-32)	42 (33-50)
Count, n	12,293	7860	4443
GS: unknown (kg)			
Score, median (IQR)	30 (22-40)	26 (20-31)	42 (32-50)
Count, n	1845	1160	685
Subset comorbidities			
Closed treatment trajectories, n	5307	3355	1952
Assigned comorbidity category, n (%)			
0	3466 (65.3)	2353 (70.1)	1113 (57.0)
1	1275 (24.0)	720 (21.5)	555 (28.4)
>1	566 (10.7)	282 (8.4)	284 (14.5)

a There are missing data for this variable: total n=64,432, women n=41,211, and men n=23,220.

b 6MWT: 6-minute walking test.

c 5TST: 5-times sit-to-stand test.

d SPPB: Short Physical Performance Battery.

e PSFS: Patient-Specific Functional Scale.

f FSS: Fatigue Severity Scale.

g GS: grip strength.

### Patient Population Receiving Physical Therapy After COVID-19

Table 1 presents the baseline scores on the outcome measures registered around the start of the physical therapy treatment trajectories for COVID-19. In 83.2% (59,184/71,152) of the treatment trajectories, at least one measurement with any of the included outcome measures at baseline was registered.

Figure 3 provides a visual representation, for women (3A) and men (3B) separately, of the relative incidence and 95% CIs of the starting treatment trajectories for COVID-19 per start week, relative to all started treatment trajectories for COVID-19 in the cohort per age category, corrected for dominant virus type at the population level (see Multimedia Appendix 2 for the model). It demonstrates a difference between the subgroups in relative incidence of starting a physical therapy treatment trajectory after COVID-19 over the first 2.5 years of the pandemic. For both men and women, the 3 older age groups (40‐59, 60‐79, and >80 y) show the greatest relative incidence of starting a physical therapy treatment after COVID-19 in mid-2021. For the younger age group (18‐39 y), the peak for both women and men is in the beginning of the fourth quartile of 2021. In line with the number of treatment trajectories per subgroup (Table 1), the CIs are widest around the oldest age group (1479 treatment trajectories for women and 1562 for men) and smallest for the age groups of 40 to 59 years (22,064 treatment trajectories for women and 10,994 for men).

**Figure 3.**
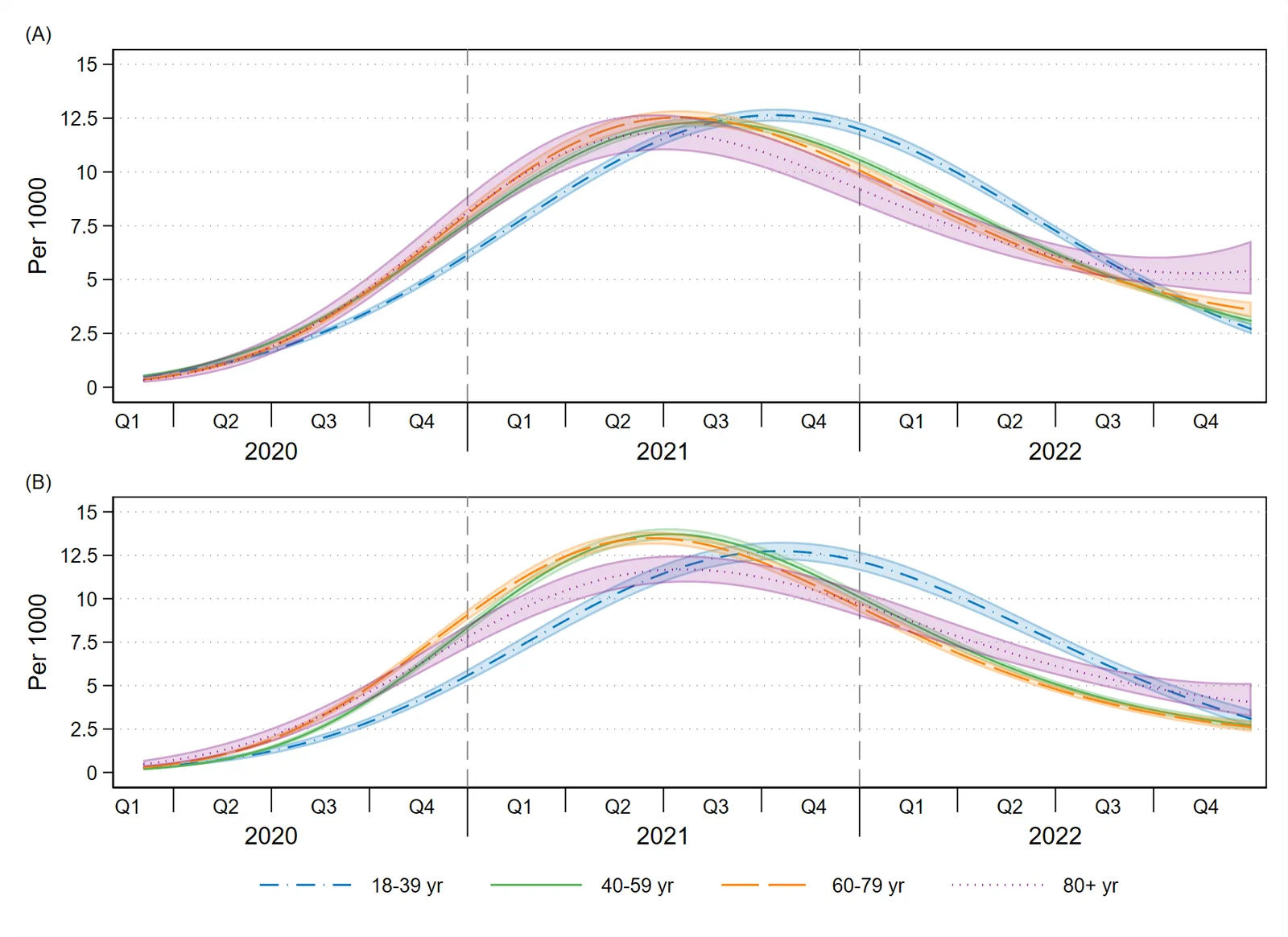
Relative incidence of the starting physical therapy treatment trajectories for COVID-19 in the Netherlands, included in the retrospective cohort over 2020 to 2022, per age category and per start week for (A) women and (B) men. Q: quartile.

### Amount of Physical Therapy Provided Within Closed Treatment Trajectories

Table 2 shows the models for the duration (in weeks) and number of consultations per closed treatment trajectory. Figure 4 displays these respective models per start week, with the 95% CIs. Overall, the closed treatment trajectories had a duration of 24 weeks with 28 consultations in the first 2.5 years of the pandemic, while correcting for age, gender, and dominant virus type (Table 2). A decreasing trend in duration (in weeks) and in number of consultations within the closed treatment trajectories was shown over time of the start of the treatment trajectories, thus patients who started a physical treatment trajectory for COVID-19 in 2020 generally received more physical therapy sessions than patients who started the treatment trajectory in 2022. For the duration of the treatment trajectory, the model shows a steeper decline at the start of the pandemic (year 2020), which stabilizes over 2021, and shows some decline again in 2022 (Figure 4). The number of consultations per treatment trajectory decreases more linearly over time (Figure 4).

**Figure 4.**
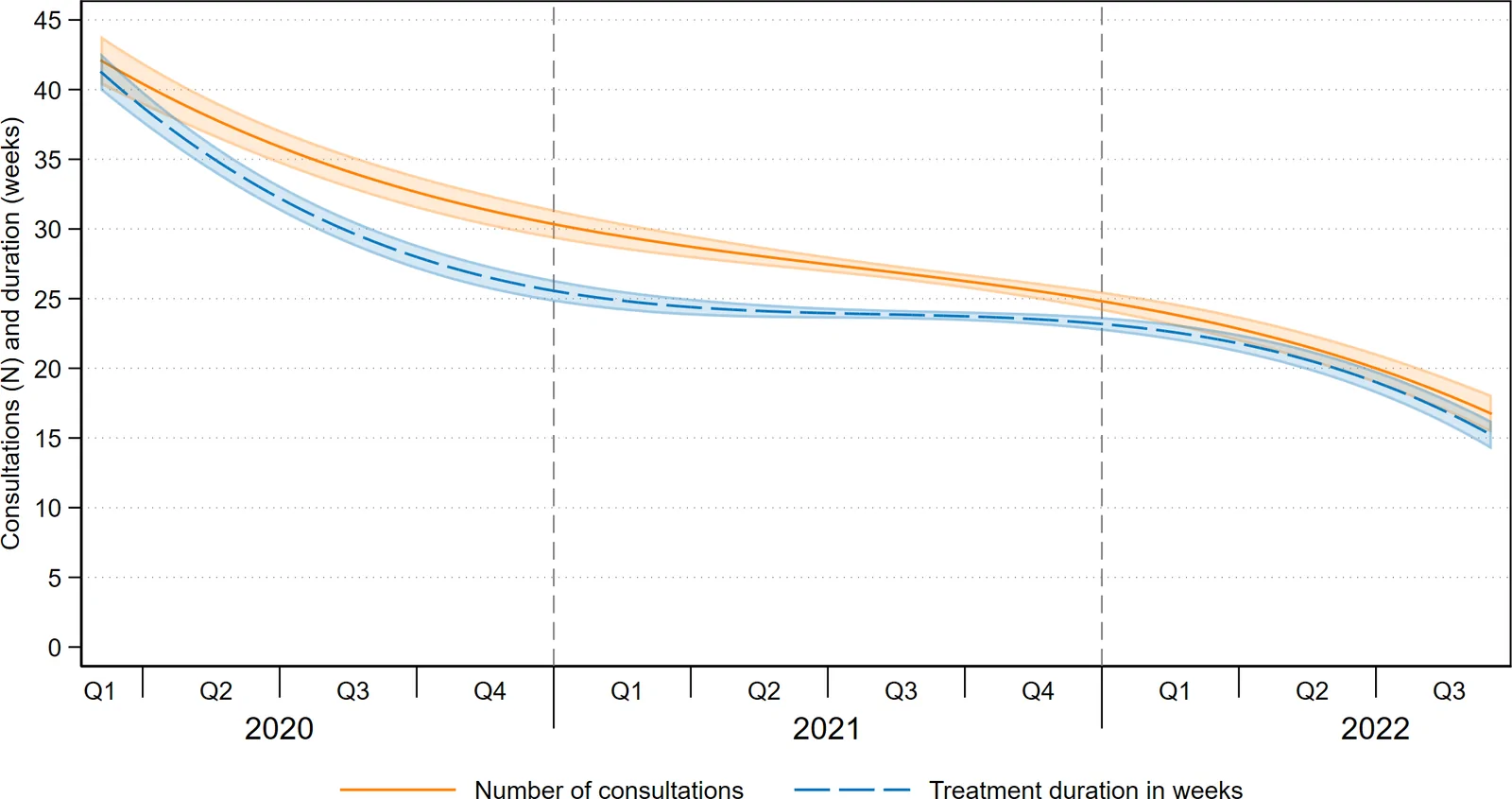
The duration (in weeks) and number of consultations in closed physical therapy treatment trajectories for COVID-19 in Dutch primary care, per start week over the period 2020 to 2022, corrected for age, gender, and dominant virus type. Q: quartile.

**Table 2.** Models for the duration (in weeks) and the number of consultations in closed physical therapy treatment trajectories for COVID-19 that started in the period 2020 to 2022 in Dutch primary care (N=66,652)^a^.

Variable	Duration (wk)		Number of consultations	
	β (SE)	*P* value	β (SE)	*P* value
Constant	24.0 (0.22)	<.001^b^	27.7 (0.32)	<.001^b^
Time	−0.022 (0.01)	.07	−0.092 (0.02)	<.001^b^
Time^2^	9.1×10^–4^ (1.1×10^–4^)	<.001^b^	3.5×10^–4^ (1.5×10^–4^)	.01
Time^3^	−4.0×10^–5^ (3.0×10^–6^)	<.001^b^	−2.3×10^–5^ (3.9×10^–6^)	<.001^b^
Age group (y)^c^				
18‐39	−1.3 (0.15)	<.001^b^	−1.9 (0.20)	<.001^b^
60‐79	0.5 (0.14)	<.001^b^	1.4 (0.18)	<.001^b^
>80	−2.6 (0.30)	<.001^b^	−4.0 (0.39)	<.001^b^
Women^d^	1.5 (0.12)	<.001^b^	0.048 (0.16)	.76
Alpha^e^	−0.6 (0.26)	.02	−0.9 (0.34)	.008^f^
Delta^e^	−2.3 (0.53)	<.001^b^	−1.0 (0.68)	.13
Omicron^e^	−3.1 (0.68)	<.001^b^	−2.7 (0.88)	.002^f^

a For one patient within this selection, information on gender was missing.

b *P*<.001.

c Patients aged 40 to 59 years were the reference group for the age variable.

d Men was the reference group for gender.

e Original Wuhan virus type was the reference group for Alpha, Delta, and Omicron.

f *P*<.01.

Both models show a significant parabolic effect of the age categories (*P*<.001) on the amount of physical therapy provided, with a maximum among older patients aged 60 to 79 years. Overall, the duration of the treatment trajectory for patients aged between 18 to 39 years was 1.3 weeks shorter than for the reference group of patients in the 40 to 59 years age group and 1.8 weeks (1.3 plus 0.5) shorter than for the 60 to 79 years age group (Table 2). Overall, the number of consultations within a treatment trajectory was also highest for the age group of 60 to 79 years, namely 1.4 consultations higher than for the reference age group of patients (40 to 59 y) and 3.3 and 5.4 consultations higher, respectively, than for patients in the age groups 18 to 39 years and 80 years and older (Table 2). Gender was only significant in the model for the duration of the treatment trajectory, with an overall 1.5-week longer duration of the treatment trajectory for women than for men (*P*<.001).

Multimedia Appendix 3 shows the results of the regression analyses for the subset of closed treatment trajectories where it was possible to correct for the number of comorbidities (5307 treatment trajectories in total). The addition of the categories was not significant for the duration of the closed treatment trajectories (one comorbidity: β 0.649, SE 0.496; *P*=.19; more than one comorbidity: β 1.265, SE 0.707; *P*=.07), nor for the number of consultations in the closed treatment trajectories (one comorbidity: β 0.869, SE 0.665; *P*=.19; more than one comorbidity: β 1.008, SE 0.947; *P*=.29).

### Registered Use of Recommended Outcome Measures

Table 3 shows the use of and the scores on the outcome measures within closed physical therapy treatment trajectories for COVID-19. Only for the PSFS, 6MWT, and grip strength were there more than 1000 baseline and posttest registrations. Overall, the PSFS was registered in 78% of the closed treatment trajectories (52,040/66,653), at baseline-only in 51% (n=33,836) and at baseline and as posttest in an additional 27% (n=18,204). A score on the 6MWT was registered as baseline in 28% (n=18,419) and as both baseline and posttest in an additional 11% (n=7316). For grip strength, this was in 15% (n=10,122) and 5% (n=3448) of the closed treatment trajectories, respectively.

**Table 3.** Usage of and scores on recommended outcome measures at baseline and at posttest in the closed physical therapy treatment trajectories for COVID-19 that started in the period 2020 to 2022 in Dutch primary care*.*

Test	Number of measurements		Scores on repeated measurements, median (IQR)		
	Baseline^a^, n	Repeated measurement, n (outliers)	Baseline	Posttest	Difference
6MWT^b^ (m)	25,735	7028 (288)	418 (349-483)	507 (440-575)	83 (40-140)
5TST^c^ (s)	2281	501	—^d^	9 (7.3‐12)	—
SPPB^e^ (points)	4429	779	—	12 (10-12)	—
PSFS^f^ (points)	52,040	18,096 (108)	8 (7-10)	1 (0‐4)	-6 (–8 to –4)
FSS^g^ (points)	897	191	—	3.7 (2.3‐5)	—
GS^h^: men (kg)					
Left	4276	1139	—	—	—
Right	4265	1135	—	—	—
Unknown	646	173	—	—	—
Selected^i^	N/A^j^	1196 (120)	40 (32-47)	45 (38-53)	5 (2-9)
GS: women (kg)					
Left	7496	1839	—	—	—
Right	7493	1838	—	—	—
Unknown	1117	268	—	—	—
Selected	N/A	2013 (119)	26 (20-30)	30 (25-35)	4 (1-7)
GS: all (kg) selected	N/A	3209 (239)	30 (22-38)	34 (27-43)	4 (2-8)
GPE^k^: recovery	—	10,736	—	2 (1-2)	—
GPE: satisfaction	—	6632	—	1 (1-2)	—

a Numbers differ from Table 1, as the numbers in this table are given within the subset of closed treatment trajectories.

b 6MWT: 6-minute walking test.

c 5TSTS: 5-times sit-to-stand test.

d Not applicable.

e SPPB: Short Physical Performance Battery.

f PSFS: Patient-Specific Functional Scale.

g FSS: Fatigue Severity Scale.

h GS: grip strength.

i Scores for chosen hand.

j N/A: not available.

k GPE: global perceived effect.

On the PSFS, where a lower score reflects improvement, the median difference score was −6 (IQR −8 to −4) points over 18,096 closed treatment trajectories (Table 3). For the 6MWT and grip strength, higher scores reflect improvement. On the 6MWT, a median difference score of 83 (IQR 40-140) m was found (n=7028). On grip strength, median difference scores of 5 (IQR 2-9) kg for men and of 4 (IQR 1-7) kg for women were found over, respectively, 1196 and 2013 closed treatment trajectories.

The GPE-recovery was registered in 10,736 closed treatment trajectories, of which 81.6% (n=8761) indicated that the recovery was “much” to “very much better” since the beginning of the treatment. In 94.4% (n=6259) of the closed treatment trajectories in which GPE satisfaction was registered (n=6632), “very” to “very much satisfaction” with the treatment was indicated.

## Discussion

### Principal Findings

This retrospective cohort study, using real-world routinely recorded data of more than 60,000 patients from EHRs of physical therapy practices, provides insight into developments in the use of physical therapy after COVID-19 in the Dutch adult population during the first 2.5 years of the pandemic. Both the relative incidence of starting physical therapy and the amount of health care provided differed between groups of age and gender. Furthermore, more than 4 out of 5 of the treatment trajectories included at least one measurement using an outcome measure that was recommended in Dutch treatment guidelines.

The retrospective cohort in this study seems representative of developments concerning coronavirus in the Netherlands during the first 2.5 years of the pandemic. For all subgroups of age and gender, the peak number of patients starting physical therapy after COVID-19 was in 2021, ranging from mid-2021 toward the end of 2021 for older age (>40 y) vs younger age groups (<40 y). These peaks are in accordance with the spread of coronavirus throughout the Netherlands, with peaks in the number of infections throughout 2021 and rapidly increasing infection rates among younger age groups, particularly with the Omicron variant. Furthermore, almost half of the treatment trajectories were for patients aged 40 to 59 years, and most of the patients were women. This is similar to characteristics of patients in a prospective study on allied health care in the Netherlands, with the inclusion period between March and June 2021, in which 64% of the participants were women and the mean age lay around 50 years [33]. Therefore, we expect that these data are representative of the provided physical therapy for COVID-19 in primary care in the Netherlands. The large sample size and use of real-world data increase the external validity of the findings.

The amount of physical therapy received was high for patients who started physical therapy after a COVID-19 infection in the beginning of the pandemic, but it rapidly decreased over the course of 2020. Notably, despite the reimbursement of 50 consultations over a period of 6 months for physical therapy after COVID-19 from the basic health insurance coverage [34], the number of consultations patients received was just over half of this number (28 consultations) for closed treatment trajectories. The latter indicates that the amount of physical therapy patients received was rapidly adjusted over time and that the possibility of declaring a large number of consultations for these patients has not been fully exploited by practitioners and patients. Notably, the amount of physical therapy received by patients after a COVID-19 infection showed a significant parabolic effect with age, with overall a maximum number of consultations and duration of the treatment trajectory among older patients aged 60 to 79 years. They had in general 3 consultations more per treatment trajectory than patients aged 18 to 39 years and 5 consultations more than patients aged 80 years or older. The number of consultations did not differ between men and women, but, on average, the treatment trajectory of women was 1.5 weeks longer, indicating a lower frequency of physical therapy for women compared to men.

The diagnostic code used in the primary physical therapy setting in the Netherlands does not allow for a differentiation between acute and postacute COVID-19, nor does it provide information on the type of symptom due to COVID-19 for which the patient sought help from the physical therapist. However, as, in the majority of the treatment trajectories, the patients visited the physical therapist after 3 to 12 weeks or after more than 12 weeks from the start of the symptoms for which the patient visited the physical therapist, the findings seem to apply primarily to patients with (early) postacute COVID-19. Additionally, the registered measures at baseline provide insight into the clinical profile at the start of the treatment trajectory of the patients visiting the physical therapist after a COVID-19 infection. In 83.2% (59,184/71,152) of the treatment trajectories, at least one measurement using any of the recommended outcome measures was registered at baseline. The baseline scores found in the present study are similar to the ones found in the nationwide prospective study, with a total of 992 patients, for the FSS [19], PSFS, 6MWT, 5TSTS, and grip strength [35]. Overall, these scores indicate severe fatigue [30], declined functional capacity by the 6MWT [36], and lower functional lower-limb strength [37] at baseline in this population, although there were no impairments in mobility according to the SPPB [38] nor weak grip strength for men or women [39].

Besides baseline measures, measures at posttest were also analyzed. In the majority of treatment trajectories (>80%), high perceived recovery and high satisfaction with the received physical therapy received were registered with the GPE. When interpreting these results, however, it is necessary to keep in mind that we made secondary use of routinely recorded health care data that were not registered for the goals of the present study. The outcome measures in the registries are not validated and the data might be biased to an extent as it is recorded in the EHRs by the therapists for different use than research. Furthermore, the lack of a control group in this study and knowledge on the specific content of the provided physical therapy does not allow for conclusions on causal relations or effectiveness. Therefore, caution is required when interpreting these measurement outcomes. There were 3 outcome measures for which a substantial sample size of closed treatment trajectories with both a baseline and posttest were available. On these, patients showed, on average, clinically relevant improvements from baseline to posttest in functional capacity (a >30.2 m increase on the 6MWT [40]) and patient-reported functional status (a >2.3 point decrease on the PSFS [41]) but not in grip strength (<5.0‐6.5 kg [29]). This is in accordance with previous studies and a recent meta-analysis showing a significant increase in the 6MWT [35,42,43] and PSFS [35] compared to a control group or to baseline, but there was no clinically relevant improvement for grip strength [35,43]. Although the baseline scores and general direction of changes from baseline to posttest are similar to the ones found in the prospective study [35], the changes reported in this study are greater. As mentioned above, it is therefore important to underline that the study design in the present study only shows improvement over time at the population level within this specific population and does not allow for a distinction between natural healing over time, the effects of possible other interventions, or the effects of physical therapy.

Notably, although most of the literature supports the use of physical exercise–based rehabilitation for persons with long COVID-19 as a potentially promising and, although symptom-dependent, effective therapy [4,43-46], there are also concerns regarding the possible detrimental effect of physical exercise. A recent systematic review and meta-analysis reported that more than half of the individuals with postacute COVID-19 syndrome perceived postexertional malaise [47], and an intensive longitudinal cohort study showed that physical activity was, among other activities, associated with subsequently increased severity of various symptoms [48]. It is therefore important that red flags, such as exertional desaturation and cardiac impairment following COVID-19, are ruled out before considering physical exercise training and that patients are assessed for postexertional symptom exacerbation, after which interventions should be modified accordingly [17,44,49]. In the present study, it is not possible to determine which patients would officially be classified as having postacute COVID-19, only that patients in the cohort were referred by a health care professional, as that was a prerequisite for reimbursement via basic health insurance.

### Limitations

There are some limitations to the present study that are important to be mentioned. First of all, the observational nature of this type of study does not allow for conclusions on causal relations or effectiveness of the health care provided. Importantly, only patients who sought physical therapy for COVID-19 at a physical therapy practice participating in one of the registries are represented in the data. Therefore, no information from a control group nor from the underlying Dutch population is available (we have no information on people who do not visit a [affiliated] physical therapist). Second, as described earlier, use of outcome measures in the registries has not been validated, and for example, it is often unknown whether a patient-reported outcome such as the PSFS or GPE is indeed answered by the patient or instead by the therapist. Third, as the amount of physical therapy provided is based on treatment trajectories that are closed, naturally the proportion of shorter closed treatment trajectories is higher near the end of the data collection. However, the impact on the current results is likely modest, as we purposefully excluded the last 22 weeks in the data for the analyses of closed treatment trajectories, and the decline is already consistently visible well before the end of the data collection. Further, the addition of registered information on comorbidities was only possible for a small subgroup, and the combination of these lower numbers and the perhaps coarse outcome of “none,” “one,” or “more than one” registered comorbidities hampers the possibility to draw conclusions based on these data. Lastly, information on other possible confounders such as social economic position or ethnic origin was not available.

### Conclusions

The present study shows the possibilities of using routinely collected health care data to gain insight into real-world physical therapy provision in primary care for COVID-19. The relative incidence of starting a physical therapy treatment trajectory for COVID-19 in primary care differed mainly between younger adults and middle-aged to older adults and was in accordance with the course of the pandemic in the Netherlands. Overall, patients showed severe perceived fatigue, declined functional capacity, and lower-limb strength at the start of the physical therapy and were satisfied with the treatment received. Furthermore, the amount of physical therapy provided declined over the first 2.5 years of the pandemic, differed between groups of age and between men and women, and was generally lower than the amount reimbursed by the Dutch government via the basic health insurance.

## Supplementary material

10.2196/88633 Multimedia Appendix 1 Definition and classification of comorbidities.

10.2196/88633 Multimedia Appendix 2 Model of relative incidence.

10.2196/88633 Multimedia Appendix 3 Models for the duration (in weeks) and number of consultations of closed treatment trajectories for the comorbidities subset.

## Abbreviations

5TSTS: 5-times sit-to-stand test
6MWT: 6-minute walking test
EHR: electronic health record
FSS: Fatigue Severity Scale
GDPR: General Data Protection Regulation
GPE: Global Perceived Effect
Nivel-PCD: Nivel Primary Care Database
PSFS: Patient-Specific Functional Scale
SPPB: Short Physical Performance Battery
