# Supplementary material for: Physical Therapy Provision After COVID-19 in the Netherlands: Retrospective Cohort Study
Source: JMIR Public Health Surveill. 2026 Sep 28;12:e88633. doi: 10.2196/88633 (PMC13618405; doi:10.2196/88633)
Supplement: Supplementary file 1 [file publichealth-v12-e88633-s001.docx]

**Appendix 1 - Defining number of comorbidities.**

Number of comorbidities was defined for patients with data available in Nivel Primary Care Database (Nivel-PCD) from general practices and/or data from registered outcome measures in the treatment-trajectory. Per treatment-trajectory was determined whether the patient was registered to have diabetes, obesity, heart failure, cardiovascular diseases and hypertension, chronic pulmonary disease, chronic kidney damage and liver disease or none of these, in at least one of these data sources. Based hereupon the patient in the treatment-trajectories with information available on comorbidities were categorized as having no comorbidity, one comorbidity or having more than one comorbidity.

*Comorbidities based on Nivel-PCD data from general practices*

For patients in the study cohort for whom also information from the electronic health records (EHRs) of their general practice was available in Nivel-PCD, data on chronic diseases registered in the EHRs by the general practitioner was used to define the presence of comorbidities for those patients. Presence of a chronic disease from the selection was based on the registered ICPC (International Classification of Primary Code) codes in the EHRs of the linked patients, with a start-date on or before the start-date of the physical therapy treatment trajectory. See table S1 for the selection of comorbidities and the accompanying ICPC-codes.

*Registered outcome measures in the treatment-trajectory*

A registration within the treatment-trajectory of the patients in the study cohort of the questionnaires ‘comorbidities’ (measurement-code 209, specs 9.0^a^) and/or ‘COVID Baseline’ (measurement-code 247, specs 9.0^a^) within a week before to one month after the start-date of the treatment-trajectory, were used to define the presence of comorbidities (table S1). In the questionnaire ‘comorbidities’ patients are asked yes/no-questions on whether they have (had) got a certain comorbidity. In the database each item represents a certain comorbidity and the score (0 or 1) is registered. In the ‘COVID-baseline’ questionnaire item 15 is used to register comorbidities and the scores represented the specific comorbidities.

| **Table S1. Selection and definition of comorbidities.** | | | |
| --- | --- | --- | --- |
| **Comorbidity** | **EHR**  **general practice** | **Registered outcome measure EHR**  **physical therapy practice** | |
|  | **ICPC code** | **“comorbidities”**  **Score 1 (=yes)** | **“COVID baseline”**  **Item 15** |
| Diabetes Mellitus | T90 | Item 1 | Score 4 |
| Overweight/obesity | - | - | Score 9 |
| Heart failure | K77 | Item 5 | - |
| Cardiovascular disease / hypertension  - Coronary diseases  - Cardiovascular accident  - Hypertension  - Narrowing of the blood vessels in the abdomen or legs (not varicose veins) | - K74, K75, K76  - K89, K90  - K85, K86, K87 | Items 3, 4, 6, 7  - 4  - 3  - 6  - 7 | Score 3 |
| Chronic lung disease  - COPD / emphysema  - Chronic bronchitis | - R95  - R91 | Item 8 | Score 2 |
| Chronic kidney disease | U99 | - | Score 5 |
| Cirrhosis of the liver | D97 | - | Score 6 |

^a^ Registration data from Electronic Health Records in physical therapy practices in the Netherlands is registered according to the specifications of that year. Outcome measures are numbered by measurement codes.

*Abbreviations:* EHR: electronic health records; ICPC: international classification of primary code; COPD: chronic obstructive pulmonary disease.
