# Supplementary material for: Physical Therapy Provision After COVID-19 in the Netherlands: Retrospective Cohort Study
Source: JMIR Public Health Surveill. 2026 Sep 28;12:e88633. doi: 10.2196/88633 (PMC13618405; doi:10.2196/88633)
Supplement: Supplementary file 2 [file publichealth-v12-e88633-s002.docx]

**Appendix 2 - Model of relative incidence.**

*Table S2.* *Model of relative incidence for the total cohort (N=71,151^a^).*

|  |  | **Total cohort** | | |
| --- | --- | --- | --- | --- |
|  |  | ß | Std. Error | *P-*value |
| Male 18-39 | Intercept | -8.6 | 0.20 | <.001^$^ |
|  | Time | 0.1 | 0.01 | <.001^$^ |
|  | Time^2 | -7.5^e-04^ | 1.0^e-04^ | <.001^$^ |
|  | Time^3 | 1.2^e-06^ | 4.0^e-07^ | .003* |
| Male 40-59 | Intercept | -9.2 | 0.12 | <.001^$^ |
|  | Time | 0.2 | 0.01 | <.001^$^ |
|  | Time^2 | -1.5^e-03^ | 6.1^e-05^ | <.001^$^ |
|  | Time^3 | 4.1^e-06^ | 2.4^e-07^ | <.001^$^ |
| Male 60-79 | Intercept | -8.7 | 0.11 | <.001^$^ |
|  | Time | 0.1 | 0.01 | <.001^$^ |
|  | Time^2 | -1.4^e-03^ | 6.3^e-05^ | <.001^$^ |
|  | Time^3 | 3.9^e-06^ | 2.5^e-07^ | <.001^$^ |
| Male 80+ | Intercept | -8.1 | 0.25 | <.001^$^ |
|  | Time | 0.1 | 0.01 | <.001^$^ |
|  | Time^2 | -1.1^e-03^ | 1.4^e-04^ | <.001^$^ |
|  | Time^3 | 3.0^e-06^ | 5.6^e-07^ | <.001^$^ |
| Female 18-39 | Intercept | -7.9 | 0.10 | <.001^$^ |
|  | Time | 0.1 | 4.2^e-03^ | <.001^$^ |
|  | Time^2 | -5.5^e-04^ | 5.4^e-05^ | <.001^$^ |
|  | Time^3 | 5.1^e-07^ | 2.2^e-07^ | .02 |
| Female 40-59 | Intercept | -8.0 | 0.07 | <.001^$^ |
|  | Time | 0.1 | 2.9^e-03^ | <.001^$^ |
|  | Time^2 | -9.1^e-04^ | 3.8^e-05^ | <.001^$^ |
|  | Time^3 | 2.1^e-06^ | 1.5^e-07^ | <.001^$^ |
| Female 60-79 | Intercept | -8.4 | 0.11 | <.001^$^ |
|  | Time | 0.1 | 4.5^e-03^ | <.001^$^ |
|  | Time^2 | -1.2^e-03^ | 5.8^e-05^ | <.001^$^ |
|  | Time^3 | 3.3^e-06^ | 2.3^e-07^ | <.001^$^ |
| Female 80+ | Intercept | -8.6 | 0.28 | <.001^$^ |
|  | Time | 0.1 | 0.01 | <.001^$^ |
|  | Time^2 | -1.4^e-03^ | 1.5^e-04^ | <.001^$^ |
|  | Time^3 | 4.4^e-06^ | 5.8^e-07^ | <.001^$^ |
| Alfa |  | 6.5^e-03^ | 0.01 | .55 |
| Delta |  | 8.7^e-03^ | 0.01 | .40 |
| Omikron |  | -3.0^e-03^ | 0.01 | .77 |
| ^*^*p*<0.01, ^$^*p*<0.001. ^a^ For 1 patient information on gender was missing. | | | | |
