# Supplementary material for: Physical Therapy Provision After COVID-19 in the Netherlands: Retrospective Cohort Study
Source: JMIR Public Health Surveill. 2026 Sep 28;12:e88633. doi: 10.2196/88633 (PMC13618405; doi:10.2196/88633)
Supplement: Supplementary file 3 [file publichealth-v12-e88633-s003.docx]

**Appendix 3 - Models for the duration (in weeks) and number of consultations of closed treatment-trajectories for the comorbidities subset.**

*Table S3.* *Models for the duration (in weeks) and number of consultations of closed treatment-trajectories for the subset of closed treatment-trajectories for which information on comorbidities was available.*

|  | **Subset for comorbidities (N=5307)** | | | | | |
| --- | --- | --- | --- | --- | --- | --- |
|  | **Duration (weeks)** | | | **Number of consultations (N)** | | |
|  | ß | Std. Error | *P-*value | ß | Std. Error | *P-*value |
| Cons | 23.0 | 0.53 | <.001^$^ | 25.9 | 0.75 | <.001^$^ |
| Time | -3.7^e-02^ | 0.04 | .40 | -0.1 | 0.06 | .03 |
| Time^2 | 4.1^e-04^ | 3.6^e-04^ | .25 | 3.6^e-04^ | 4.8^e-04^ | .45 |
| Time^3 | -3.0^e-05^ | 1.1^e-05^ | .005* | -1.4^e-05^ | 1.4^e-05^ | .31 |
| Age 20-39 | -1.6 | 0.51 | .001* | -2.2 | 0.68 | .001* |
| Age 60-79 | -1.1 | 0.50 | .04 | 0.1 | 0.68 | .89 |
| Age 80+ | -2.4 | 1.07 | .03 | -4.7 | 1.43 | <.001^$^ |
| Female | 1.1 | 0.41 | .007* | -0.5 | 0.55 | .33 |
| Alfa | -0.4 | 0.91 | .62 | -1.4 | 1.19 | .23 |
| Delta | -1.3 | 1.81 | .47 | -3.1^e-02^ | 2.37 | .99 |
| Omicron | -1.9 | 2.27 | .40 | -1.8 | 2.99 | .55 |
| Comorb 1 | 0.6 | 0.50 | .19 | 0.9 | 0.67 | .19 |
| Comorb >1 | 1.3 | 0.71 | .07 | 1.0 | 0.95 | .29 |
| ^*^*P*<.01, ^$^*P*<.001. | | | | | | |
